# Mechanism of catalysis and inhibition of *Mycobacterium tuberculosis* SapM, implications for the development of novel antivirulence drugs

**DOI:** 10.1038/s41598-019-46731-6

**Published:** 2019-07-16

**Authors:** Paulina Fernandez-Soto, Alexander J. E. Bruce, Alistair J. Fielding, Jennifer S. Cavet, Lydia Tabernero

**Affiliations:** 1 School of Biological Sciences, Faculty of Biology Medicine and Health, University of Manchester, Manchester Academic Health Science Centre, Manchester, M13 9PT UK; 2 Pharmacy and Biomolecular Sciences, Liverpool John Moores University, James Parsons Building, Byrom Street, Liverpool, L3 3AF UK

**Keywords:** Enzyme mechanisms, Enzymes

## Abstract

*Mycobacterium tuberculosis* (Mtb) SapM is a secreted virulence factor critical for intracellular survival of the pathogen. The role of SapM in phagosome maturation arrest in host macrophages suggests its potential as a drug target to assist in the clearance of tuberculosis infection. However, the mechanism of action of SapM at the molecular level remains unknown. In this study, we provide new insights into the mechanism of catalysis, substrate specificity and inhibition of SapM, and we identify the critical residues for catalysis and substrate binding. Our findings demonstrate that SapM is an atypical monoester alkaline phosphatase, with a serine-based mechanism of catalysis probably metal-dependent. Particularly relevant to SapM function and pathogenesis, is its activity towards PI(4,5)*P*_2_ and PI3*P*, two phosphoinositides that function at the early stages of microbial phagocytosis and phagosome formation. This suggests that SapM may have a pleiotropic role with a wider importance on Mtb infection than initially thought. Finally, we have identified two inhibitors of SapM, L-ascorbic acid and 2-phospho-L-ascorbic, which define two different mechanisms by which the catalytic activity of this phosphatase could be regulated. Critically, we demonstrate that 2-phospho-L-ascorbic reduces mycobacterial survival in macrophage infections, hence confirming the potential of SapM as a therapeutic drug target.

## Introduction

Tuberculosis (TB), caused by infection with *Mycobacterium tuberculosis* (Mtb), is one of the leading causes of death and poverty worldwide. The rise of multi-drug resistant (MDR), rifampicin resistant (RR), and extensively drug-resistant (XDR) TB poses major challenges in the treatment and eradication of this disease, which claims over 1.6 million lives every year (WHO TB Report 2017).

Mtb is an intracellular pathogen that prevents its clearance in the host by blocking critical phagosome maturation events inside alveolar macrophages^1–3^. Subversion of phosphoinositide (PI) metabolism and dynamics is a key mechanism used by Mtb and other bacterial pathogens to arrest phagolysosome fusion thus preventing bacterial killing^4,5^. PIs are essential for the recruitment of effector proteins like EEA1 and the membrane remodelling ESCRT complexes, which drive the progression of early phagosomes to the degradative lysosomal pathway to eliminate the pathogen^5^. For Mtb infection, two secreted phosphatases have been implicated in subverting PI metabolism in the host to promote intracellular survival, MptpB^6–8^ and SapM^9,10^.

SapM, was initially described as a nonspecific acid phosphatase^11^, and later reported to dephosphorylate PI3*P*^9^ a central player in phagosomal maturation^12,13^. Studies involving *sapM* deficient Mtb mutants supported a role for this protein in phagosome maturation arrest^10,14^ by preventing Rab5-Rab7 exchange^15^. In addition, SapM appears to have a role in immunogenicity^14,16,17^ and autophagy inhibition^18^. Notably, deletion of the *sapM* gene attenuated Mtb intracellular growth in human and mouse macrophages^10,14^ and reduced the mycobacterial burden in guinea pig models of infection^10^. However, deletion of *sapM* did not affect mycobacterial growth in extracellular cultures^10^ thus confirming its role as a virulence factor important for intracellular mycobacterial survival.

The growing evidence on the importance of SapM in pathogenesis makes it an attractive drug target for TB therapy, particularly since antivirulence drugs are gathering momentum as new therapeutic approaches to fight antimicrobial resistance^8,19–21^. However, the molecular mechanism of catalysis and mode of action of SapM is still largely unknown. This knowledge is crucial to fully exploit its potential as a pharmacological target, and to guide the design of specific inhibitors for drug development. To date a full biochemical and kinetics characterisation of this phosphatase has not been possible due to challenges in producing recombinant SapM. We have now successfully expressed SapM in *E*. *coli* and purified it to homogeneity for enzymatic profiling and inhibition studies. The enzyme kinetics analysis and mutagenesis of conserved residues, indicates that SapM follows a serine-based mechanism of catalysis similar to alkaline phosphatases. The substrate specificity and inhibition profiles for SapM show differences with classical alkaline phosphatases indicating that SapM is an *atypical* member of this family.

In addition, we provide evidence of SapM activity towards a wide range of PIs with preference for PI(4,5)*P*_2_ and PI3*P*. This suggests a potential new role for this phosphatase in pathogenesis by acting at the early stages of phagocytosis as well as in phagosomal maturation. Finally, we report that 2-phospho-L-ascorbic acid (2P-AC) is a competitive inhibitor of SapM and that reduces intracellular survival of Mtb in infected macrophages. This is the first evidence that inhibition of SapM could be exploited to control Mtb infection and underlines the potential for anti-virulence approaches to develop new therapies to treat TB.

## Results

### SapM shares conserved functional residues with acid and alkaline phosphatases

**Figure 1:**
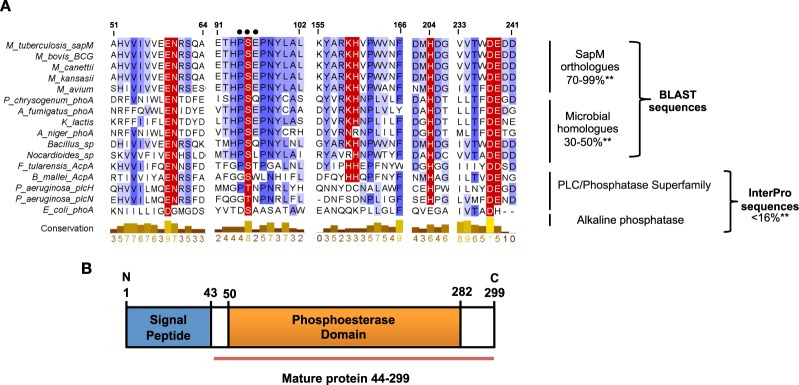
Sequence alignment and domain architecture of SapM. (**A**) Multiple sequence alignment showing the active site region of SapM orthologues and related sequences. Alignment is shaded blue according to sequence conservation (conservation is also given by numbers from 1 to 10, with 10 (*) being 100% conservation). Numbers above the alignment correspond to SapM residue numbering. The catalytic residues are denoted on red background with white letters. Gene names are included alongside with microbial pathogen names. Alignment was performed using Clustal Omega^58^ and figure with Jalview Version 2.10.3b1^59^. Two asterisks denote pairwise identity. Black dots denote the catalytic motif in both SapM orthologues (PSE) and alkaline phosphatase (DSA). (**B**) Domain architecture of SapM with boundaries for the mature protein annotated.

Bioinformatics approaches were used to analyse the sequence of SapM and related proteins. A BLAST search revealed orthologues of SapM in all mycobacterial species with 70–99% pairwise identity (Fig. 1A). SapM-related sequences were also found in other bacterial and fungal organisms, although with lower identity (30–50%). Most of these proteins are currently annotated either as hypothetical proteins, uncharacterised or acid phosphatases, but their catalytic mechanism is unknown. These searches did not identify related sequences in higher eukaryotes.

InterPro analysis showed that SapM possesses a signature match to the *phosphoesterase domain* that is present in the superfamily of alkaline phosphatases^22,23^ and in the superfamily of acid phosphatases and phospholipases C (PLC/phosphatase superfamily)^24^.A prototype of classic alkaline phosphatases (AlkPs) is the *Escherichia coli* alkaline phosphatase (Ec-AlkP)^25^, which shares 23% sequence similarity (16% identity) to SapM (Fig. 1A). Likewise, a well-characterised member of the PLC/phosphatase superfamily, the *Francisella tularensis* AcpA (Ft-AcpA), with a 3D structure similar to alkaline phosphatases^26^, shares 25% sequence similarity (15% identity) to SapM.

The low pairwise identity values made difficult a direct assignment of this enzyme to any of these families, but a multiple sequence alignment with representatives across the different superfamilies and the SapM-related sequences (Fig. 1A) highlighted the conservation within the *phosphoesterase domain*, of residues essential for catalysis and substrate binding in Ft-AcpA^26,27^, and Ec-AlkP^25^. This conservation includes the nucleophile serine (S175 in Ft-AcpA and S102 in Ec-AlkP), metal binding and substrate binding residues (Fig. 1A). Thus, the analysis suggested that SapM may share a similar serine-based, potentially metal-dependent, mechanism of catalysis with these enzymes, despite the modest overall conservation. To elucidate the catalytic mechanism of SapM, we carried out a comprehensive biochemical and kinetic characterisation of the enzyme.

### The optimal enzymatic activity for SapM is at a basic pH

**Figure 2:**
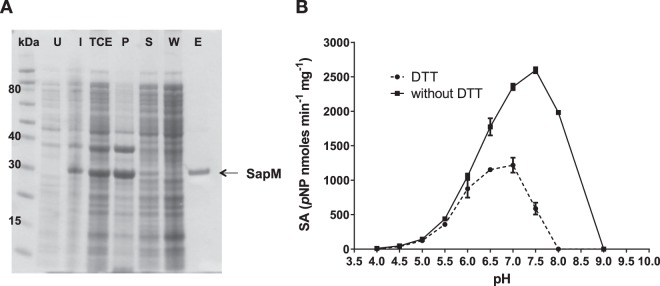
Expression, purification and enzymatic activity of SapM. (**A**) Coomassie stained 10% SDS-PAGE gel shows expression and purification of recombinant SapM (Mw 28.8 kDa) (lane E). Solubilisation was achieved using 1% sarkosyl in the lysis buffer. Purification was done by nickel-affinity chromatography and eluted with 200 mM imidazole. U: uninduced, I: induced, TCE: total cell extract, P: pellet, S: soluble, W: wash, and E: elution. (**B**) Enzymatic activity of SapM was assessed using the *p*-Nitrophenyl phosphate (*p*NPP) assay. Hydrolysis of *p*NPP was measured at pH values ranging from 4 to 9 in the presence and absence of 3 mM dithiothreitol (DTT). Specific activity (SA) was calculated as nanomoles of *p*-nitrophenol (*p*NP) released per mg of protein and min of the reaction. Error bars represent standard deviation of the mean (SD) of triplicates.

The SapM mature secreted native protein comprises residues 44 to 299^11^, which contains the whole phosphoesterase domain (Fig. 1B). A construct with a His-tagged version of the mature sequence was expressed in *E*. *coli* C41 (DE3), taking advantage of the periplasmic localisation sequence provided by the pET22b vector (Novagen). Soluble SapM was successfully obtained after treatment of the bacterial cell pellet with the ionic surfactant sarkosyl^28^ (see methods for details). Subsequently, purification by nickel-affinity chromatography produced 95% pure protein (Fig. 2A). SapM migrates according to the predicted molecular weight of 28.8 kDa for the mature protein plus the His_6_-tag (as calculated in ProtParam^29^). The identity of the purified protein was confirmed by tryptic digestion followed by LC/MS/MS analysis, and the intact mass calculated as 28,772.3 ± 0.19 Da. To the best of our knowledge, this is the first report of SapM being produced as a soluble recombinant protein following expression in *E*. *coli*.

Next, we evaluated the enzymatic activity of the purified SapM protein by measuring the conversion of *p*-Nitrophenyl phosphate (*p*NPP) into *p*-nitrophenol at different pH values and in the presence or absence of dithiothreitol (DTT). The optimal activity for SapM was obtained at pH 7.5 in the absence of DTT (Fig. 2B). We also confirmed the optimal activity at this pH for the protein after cleavage of the His-tag (Suppl. Fig. 1). This is in agreement with the enzyme activity pH range (6.5–7.5) reported for the native SapM^11^.

The addition of 3 mM DTT resulted in a considerable reduction of the specific activity at any pH, with a drop of activity of about 70% at pH 7.5 (Fig. 2B). Usually, DTT is used to prevent disulfide bond formation by reduction of Cys residues, which is particularly critical in Cys-based phosphatases where oxidation of the catalytic cysteine abolishes enzyme activity^30^. However, SapM protein lacks the hallmark active site P-loop motif (CX5R) present in Cys-based phosphatases^31^. Therefore, the effect of DTT potentially suggests a different mechanism in reducing enzyme activity. DTT can also act as a metal chelator due to its ability to form stable coordination complexes with metals^32,33^. Since the metal-binding residues identified in Ft-AcpA and Ec-AlkP are conserved in SapM, a potential explanation would be that the effect of DTT is due to chelation of a bound metal. Alternatively, DTT may impact on a metal oxidation state, which could similarly result in metal dissociation. These findings are therefore consistent with the suggestion that SapM is also a metal-dependent enzyme.

### Mutagenesis of conserved residues suggests an alkaline-like mechanism of catalysis

**Figure 3:**
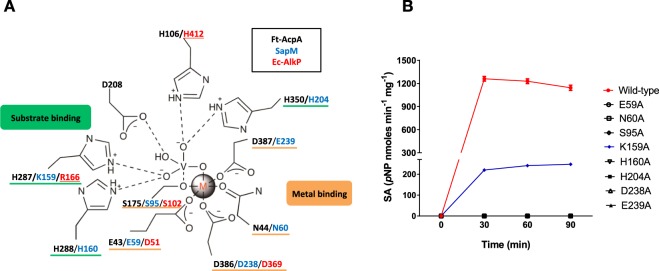
Identification of catalytic residues in SapM. (**A**) Schematic diagram showing the active site residues of AcpA from *F*. *tularensis* (Ft-AcpA in black). Conserved residues in SapM (in blue) and in the alkaline phosphatase from *E*. *coli* (Ec-AlkP in red) are shown. The metal ion has an octahedral coordination made of oxygens in AcpA and the vanadate that occupies the substrate-binding site^26^. The underlined residues from Ec-AlkP occupy similar structural location with Ft-AcpA. SapM residues involved in metal binding (underlined in orange) and substrate binding (underlined in green) are highlighted. (**B**) Specific Activity (SA) of wild-type and mutants of the conserved residues in SapM measured by *p*NPP assay. Mutant K159A shows reduced activity with respect to the wild-type whereas the other mutations completely abolish the enzymatic activity of SapM (values overlap along the X-axis). Specific activity was calculated as nanomoles of *p*-nitrophenol (*p*NP) released per mg of protein and min of the reaction. Error bars indicate standard error of the mean (SEM) of two independent experiments.

Our bioinformatics analysis showed that SapM shares conserved catalytic residues with Ec-AlkP and Ft-AcpA (Fig. 1A), both of which also follow an alkaline phosphatase mechanism of catalysis^25,26^. Ft-AcpA and Ec-AlkP both use a serine (S175 in Ft-AcpA and S102 in Ec-AlkP) as the nucleophile, which is activated by metal ions, three in the case of Ec-AlkP (two Zn^2+^ and Mg^2+^)^25,34^ and only one in the case of Ft-AcpA^26^. The crystal structure of Ft-AcpA in complex with vanadate^26^ revealed four histidines (H106, H287, H288 and H350) critical for substrate binding. In the Ft-AcpA structure the metal ion is coordinated by seven oxygen atoms from residues N44, E43, D386, D387 and the vanadate molecule (Fig. 3A). Key catalytic residues in Ft-AcpA are also present in the structure of Ec-AlkP^25^ (Fig. 3A). Based on the multiple sequence alignment, up to eight residues important in Ft-AcpA catalysis and substrate binding are conserved in SapM (Figs 1A, 3A).

We explored the role of these conserved residues in SapM, by mutagenesis and enzyme activity assays. The SapM mutants S95A, N60A, E59A, D238A, E239A, H160A and H204A exhibited no activity towards *p*NPP, even at longer incubation times of up to 90 min (Fig. 3B). Using the catalytic mechanism described for Ft-AcpA as a reference, we propose S95 to act as the nucleophile in SapM. Residues E59, N60, D238 and E239 align with the metal-binding site described for Ft-AcpA, and an analogous role in metal-binding is supported by their requirement for SapM-mediated catalysis. The residues H160 and H204 may be involved in substrate binding as their cognate residues H288 and H350 in Ft-AcpA. In fact, mutation of Ft-AcpA H288 and H350 to alanine, also resulted in lack of enzyme activity in that enzyme^27^.

Interestingly, the SapM K159A mutant showed only reduced activity towards *p*NPP with respect to the wild-type enzyme (Fig. 3B), indicating that it is important but not essential for catalysis. Analogous residues H287 in Ft-AcpA^27^ and R166 in Ec AlkP^25^, appear to have a role in substrate binding as well. Our mutagenesis studies thus confirmed the importance of the conserved residues S95, N60, E59, D238, E239, H160, H204 and K159 in SapM catalysis and substrate binding. These data and the proposed role for these residues indicate that SapM follows a serine-based alkaline-like mechanism of catalysis similar to that of Ec-AlkP and Ft-AcpA with S95 acting as the nucleophile.

### Influence of metal ions in SapM enzymatic activity

**Figure 4:**
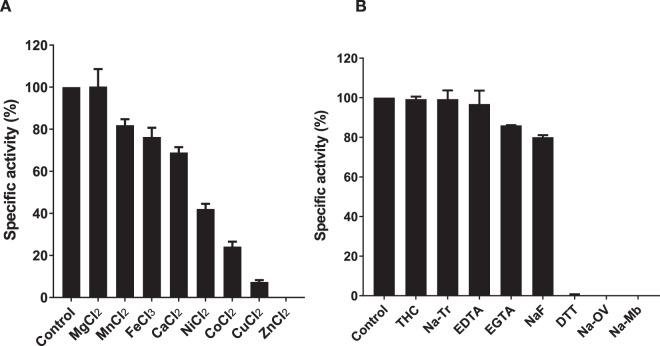
Effect of metals and inhibitors on the enzymatic activity of SapM. Enzymatic activity of SapM was assessed using the *p*NPP assay. (**A**) SapM activity is tested in the presence of various metals (10 mM, except for FeCl_3_ that was used at 1 mM). (**B**) SapM activity was tested in the presence of 10 mM of metal chelators ethylenediaminetetraacetic acid (EDTA) egtazic acid (EGTA) and inhibitory compounds: orthovanadate (Na-OV) and sodium molybdate (Na-Mb), tetramisole hydrochloride (THC), sodium tartrate (Na-Tr), sodium fluoride (NaF) and dithiothreitol (DTT). Percentage of specific activity is calculated relative to the amount of *p*-nitrophenol released from the hydrolysis of *p*NPP of the control (without metals or inhibitors). Error bars in A and B indicate SEM of two independent experiments.

In order to study the potential role of metal ions in SapM activity, we performed enzymatic assays in the presence of a range of transition (Co^2+^, Ni^2+^, Zn^2+^, Mn^2+^, Cu^2+^and Fe^3+^) and alkaline metals (Ca^2+^ and Mg^2+^). The activity of SapM was totally inhibited by zinc, and reduced by >50% in the presence of copper, cobalt and nickel. Magnesium was the only metal that did not affect the enzymatic activity of SapM (Fig. 4A). Next, using electron paramagnetic resonance (EPR) spectroscopy, we tested for the presence of Fe^3+^, Ni^2+^, Mn^2+^, Cu^2+^ and Co^2^^+^. No signal for any of these metal ions was detectable, indicating that none of these metals are bound to the enzyme under the conditions tested. We also carried out inductively coupled plasma mass spectrometry (ICP-MS) analysis of purified SapM, which failed to detect the presence of protein associated metals (Mg, Ca, Cr, Co, Ni, Cd, Cu, Zn or Fe). Thus, whilst none of these metals are co-purified with SapM, it remains possible that they can bind to SapM and inhibit activity by displacing an as yet unidentified metal-cofactor. It is noteworthy that these experiments were repeated with SapM purified with EDTA-free buffers (including lysis buffer), but the results were unchanged.

High concentrations of DTT (10 mM) completely inhibited SapM activity (Fig. 4B), supporting the idea that DTT may be acting (chelating or changing oxidation state) on an unknown metal that is resistant to high concentrations (10 mM) of the divalent chelators ethylenediaminetetraacetic acid (EDTA) and egtazic acid (EGTA) (Fig. 4B).

### SapM is an atypical monoester alkaline phosphatase

**Figure 5:**
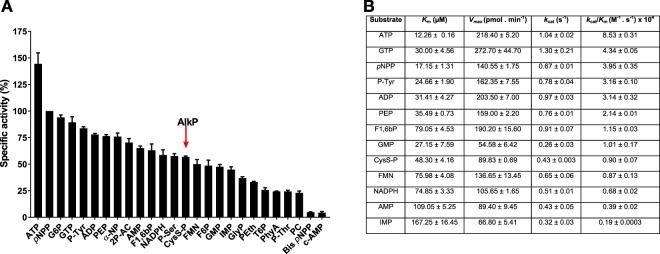
Substrate specificity profile for SapM. Enzymatic activity of SapM protein was assessed using the malachite green assay. (**A**) SapM was tested for hydrolysis of phosphosubstrates at 80 µM. Percentage of specific activity is calculated relative to the amount of inorganic phosphate (P_i_) released from the hydrolysis of *p*NPP substrate. Arrow highlights a substrate specific for alkaline phosphatases (AlkP). Error bars indicate SEM of three independent experiments. (**B**) Kinetic parameters for SapM dephosphorylation of substrates with specific activity >50% with respect to *p*NPP substrate. Values indicate SEM of two independent experiments. ATP (adenine triphosphate), G6P (glucose 6-phosphate), GTP (guanosine triphosphate), P-Tyr (phosphotyrosine), ADP (adenosine diphosphate), PEP (phosphoenolpyruvate), α-NP (α-Naphthyl phosphate), 2P-AC (2-phospho ascorbic acid), AMP (adenosine monophosphate), F1,6bP (fructose 1,6-bisphosphate), NADPH (nicotinamide adenine dinucleotide 2′-phosphate reduced), P-Ser (phosphoserine), CysS-P (cysteamine *S*-phosphate), FMN, (riboflavin monophosphate), F6P (fructose 6-phosphate), GMP (guanosine monophosphate), IMP (inosine monophosphate), GlyP (glycerophosphate), PEth (O-phosphorylethanolamine), T6P (threalose 6-phosphate), PhyA (phytic acid), P-Thr (phosphothreonine), PC (Phosphorylcholine chloride), Bis *p*NPP (bis *p*-nitrophenyl phosphate), c-AMP (adenosine 3′,5′-cyclic monophosphate).

Next, we tested a wide range of phospho-substrates to establish the specificity of SapM. SapM hydrolysed a wide range of different phospho-substrates and fifteen of them with a specific activiy of at least 50% with respect to *p*NPP (Fig. 5A), indicating a broad specificity. Kinetic analysis of the best substrates showed that SapM displays the highest efficiency (*k*_*cat*_/*K*_*m*_) towards the triphosphate nucleosides ATP and GTP (Fig. 5B). SapM also hydrolysed cysteamine *S*-phosphate, a substrate for classic alkaline phosphatases^35^, with a specific activity of 57% with respect to *p*NPP hydrolisis (Fig. 5A).

The activity of SapM towards the phosphodiester substrates bis-*p*-nitrophenyl phosphate (Bis *p*NPP) and adenosine 3′,5′-cyclic monophosphate (c-AMP), typical substrates of phosphodiesterases^36,37^, was less than 5% with respect to *p*NPP hydrolisis. SapM therefore shows a clear preference for phosphomonoesters over phosphodiester substrates, ruling out that it functions as a phosphodiesterase.

Next we tested common inhibitors of protein phosphatases. The oxyanions, vanadate and molybdate, which are inhibitors of classic phosphotyrosine phosphatases (PTPs)^38^, abolished SapM activity (Fig. 4B). High concentrations (10 mM) of tetramisole hydrochloride, an alkaline phosphatase inhibitor^35^, did not inhibit the enzyme activity. Likewise, SapM activity was resistant to sodium fluoride (acid phosphatase inhibitor) and sodium tartrate (acid phosphatase and Ser/Thr phosphatase inhibitor) (Fig. 4B). These findings suggest a mixed type of inhibition profile for SapM matching neither classic acid phosphatases, nor alkaline phosphatases.

The broad substrate specificity of SapM, together with the optimal pH of 7.5 is similar to that observed in the alkaline phosphatase family. However, SapM shares only 16% identity with the closest classical alkaline phosphatase, Ec-AlkP, and lacks the characteristic catalytic motif present in alkaline phosphatases, *DSA* (D101, S102 and A103 in Ec-AlkP), where S is the nucleophile residue^25^. Instead, SapM has a *PSE* motif, conserved in all mycobacterium orthologues and microbial related sequences (Fig. 1A), which contains the catalytic S95.

Previous studies suggested that SapM could be a nonspecific acid phosphatase (NSAP) or a His-based phosphatase^11^. Our analyses indicate that SapM does not contain the characteristic motifs found in His-based phosphatases, such as the catalytic R**H**GXRXP motif^39^, where the histidine is the nucleophile. SapM is also missing the conserved active site motif CX_5_R of acid phosphatases^31^, and does not present any of the characteristic motifs of NSAP described up to now^40^. These data, together with the identification of the catalytic residues, indicate that SapM behaves as an atypical alkaline phosphatase with broad monoesterase activity.

### SapM shows specificity for PI(4,5)P_2_ and PI3P

**Figure 6:**
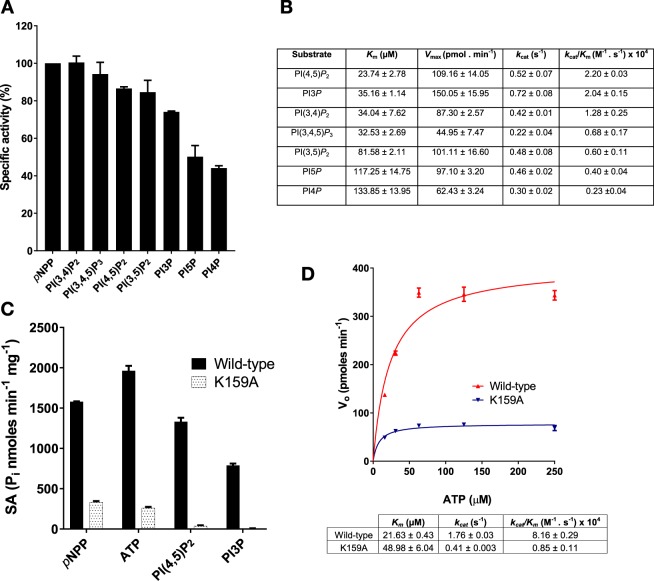
SapM hydrolyses phosphoinositides (PIs). Enzymatic activity of SapM protein was assessed using the malachite green assay. (**A**) SapM activity was tested on seven phosphoinositides at 80 µM. Percentage of specific activity is calculated relative to the amount of inorganic phosphate (P_i_) released from the hydrolysis of *p*NPP substrate. (**B**) Kinetic parameters for SapM dephosphorylation of phosphoinositides. PI(3,4)*P*_2_ (phosphoinositide 3,4 di-phosphate), PI(3,4,5)*P*_3_ (phosphoinositide 3,4,5 tri-phosphate), PI(4,5)*P*_2_ (phosphoinositide 4,5 di-phosphate), PI(3,5)*P*_2_ (phosphoinositide 3,5 di-phosphate), PI3*P* (phosphoinositide 3 phosphate), PI4*P* (phosphoinositide 4 phosphate), PI5*P* (phosphoinositide 5 phosphate). (**C**) The enzymatic activity of K159A mutant was tested on *p*NPP, ATP, PI(4,5)*P*_2_ and PI3*P* at 80 µM. Specific activity was calculated as nanomoles of inorganic phosphate released per mg of protein and min of the reaction. (**D**) Kinetic analysis of the K159A towards ATP respect to the wild-type enzyme. Values and error bars in A, B, C and D indicate SEM of two independent experiments.

Previous studies^9^ suggested that the native SapM was able to dephosphorylate mono-phosphorylated phosphoinositides (PIs), specifically PI3*P*. However, nothing was reported about SapM activity towards the di and tri-phosphorylated PIs. Surprisingly, the isolated SapM can hydrolyse a wide range of phosphoinositides (Fig. 6A), but preferentially dephosphorylates PI(4,5)*P*_2_ and PI3*P*, showing higher *k*_*cat*_/*K*_*m*_ values over the rest. In contrast, SapM shows 5 to 10 times lower catalytic efficiency towards the mono-phosphorylated PI4*P* and PI5*P* due to a much lower affinity (*K*_*m*_) for these compounds (Fig. 6B).

Interestingly, the K159A mutant showed less than 2% activity towards PI(4,5)*P*_2_, and PI3*P* whilst retaining 21% and 13% activity for *p*NPP and ATP, respectively (Fig. 6C). Kinetic analyses also revealed a 2-fold increase in *K*_*m*_ and a 10-fold decrease in *k*_*cat*_/*K*_*m*_ for K159A towards ATP (Fig. 6D), confirming its role in substrate binding as proposed for its analogues H287 in Ft-AcpA^27^ and R166 in Ec-AlkP^41^. Hence, similarly to R166 in Ec-AlkP^25^, K159 may provide additional stabilisation of the negatively charged phosphosubstrates, which is clearly critical for hydrolysis of phosphoinositides that contain extra negatively charged phosphate groups.

The specificity of SapM for PI(4,5)*P*_2_ in addition to PI3*P* is intriguing and suggests that this phosphatase may have more than one role in infection and pathogenesis, since these PIs localise to the plasma/phagocytic membrane and early phagosome membrane, respectively.

### Inhibition of SapM enzymatic activity

**Figure 7:**
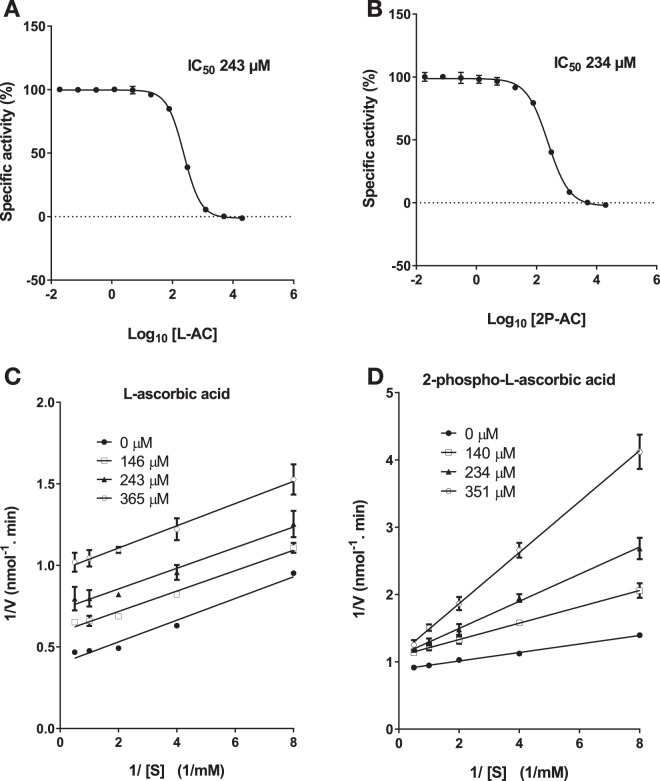
Mechanism of inhibition of SapM activity by L-ascorbic acid and 2-phospho-L-ascorbic acid. Inhibition curves for L-ascorbic acid (L-AC) (**A**) and 2-phospho-L-ascorbic acid (2P-AC) (**B**) using the *p*NPP assay. Percentage of specific activity is calculated relative to the amount of *p*-nitrophenol released in the absence of inhibitor. Lineweaver-Burk plots of SapM activity towards *p*NPP at increasing concentrations of L-AC (**C**) and 2P-AC (**D**). L-AC behaves as uncompetitive inhibitor as noted by variations of the *V*_*max*_ and the *K*_*m*_ at different inhibitor concentrations, whereas 2P-AC behaves as a competitive inhibitor as observed by changes of the *K*_*m*_ while the *V*_*max*_ remained unchanged at different inhibitor concentrations. Error bars in A, B, C and D represent SD of triplicates.

Inhibition studies indicated that L-ascorbic acid (L-AC) and 2-phospho-L-ascorbic acid (2P-AC) inhibit the enzymatic activity of Ft-AcpA^27^. Since SapM shares a similar mechanism of catalysis with Ft-AcpA, we speculated that these compounds may also inhibit SapM. An initial test showed that 10 mM of L-AC and 2P-AC completely abolished the enzyme activity of SapM. Subsequently we determined the IC_50_ to be 241 µM for L-AC and 234 µM for 2P-AC (Fig. 7A,B). The mechanism of inhibition by these compounds differs. L-AC acts as uncompetitive inhibitor as noted by variations of the *V*_*max*_ and the *K*_*m*_ at different inhibitor concentrations (Fig. 7C). L-AC is a potent oxidant and thus its inhibition may be the result of metal oxidation in the enzyme-*p*NPP complex^42^, explaining the uncompetitive mechanism of inhibition observed. In contrast, 2P-AC behaves as a competitive inhibitor, since variations in its concentration produced marked changes in the *K*_*m*_ while the *V*_*max*_ remained unchanged (Fig. 7D). Since SapM can also hydrolyse 2P-AC (Fig. 5), this compound may inhibit SapM activity in a two-way mechanism: by competing with *p*NPP for binding to the active site, and as a metal oxidant, as a result of its ascorbic acid production due to hydrolysis.

### 2-phospho-L-ascorbic acid reduces survival of *M. tuberculosis* in infected macrophages, although does not affect extracellular growth

**Figure 8:**
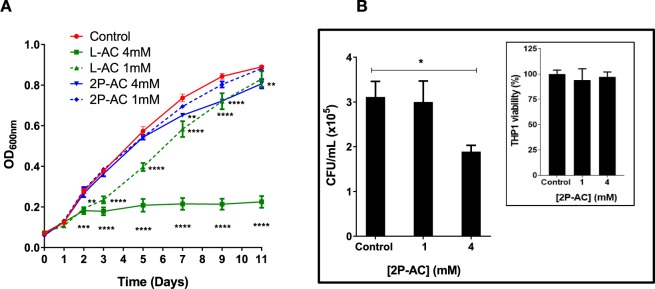
Effect of L-AC and 2P-AC on *Mycobacterium tuberculosis* H37Rv growth. (**A**) The inhibitory effect of L-AC and 2P-AC on the extracellular growth of *M*. *tuberculosis* (Mtb) H37Rv was monitored by measuring optical density (OD_600_) over time. Cultures were treated with 1 mM or 4 mM of the inhibitors at day 0 and 1 and grown over 11 days. Statistical significance was evaluated by two-way ANOVA (Bonferroni test) where **p = 0.0039; **p = 0.0048; **p = 0.0059; ***p = 0.0010 and ****p < 0.0001. Control is without inhibitor. (**B**) Effect of 2P-AC on intracellular Mtb survival in human THP1 macrophages. Significant reduction of mycobacterial growth (*p = 0.0365 by one-way ANOVA, Bonferroni test) is observed at 4 mM compared to the control (without inhibitor). Plots represent the average of CFU/ml at 72 h post infection. Inset shows THP1 viability at 72 h upon treatment with 2P-AC. Error bars in A and B indicate SEM of two independent experiments.

Next, we evaluated the effect of these SapM inhibitors on Mtb extracellular growth. For this, growth of the Mtb H37Rv strain was monitored over the course of 11 days (Fig. 8A) with and without the addition of the inhibitors. Cultures were treated with different concentrations of L-AC and 2P-AC added in day 0 and 1, and growth was monitored using optical density (OD_600_). Mycobacterial growth was severely reduced from day 2 with 4 mM L-AC, and even a lower concentration (1 mM) L-AC had a significant (p = 0.0039) effect in growth reduction (Fig. 8A). These findings are in agreement with previous studies showing the effect of L-AC on mycobacterial growth^43^. In contrast, treatment with 1 mM 2P-AC did not affect mycobacterial growth (Fig. 8A), while treatment with 4 mM 2P-AC showed only significant reduction of mycobacterial growth from day 7 (p = 0.0048). The delayed effect of 2P-AC may be due to slow spontaneous hydrolysis in solution of the phosphate group to generate ascorbic acid, as previously reported^44^, plus hydrolysis by the secreted SapM. Thus, while L-AC shows a severe effect on inhibiting Mtb extracellular growth, the presence of the phosphate group in 2P-AC appears to have a protective effect, during the first 7 days post-treatment.

Previous studies have demonstrated that deletion of the *sapM* gene, results in reduction of mycobacterial burden within macrophages and guinea pig models of infection^10,14,15^. We hypothesised that chemical inhibition of SapM may recapitulate the effect of the *sapM* knockout, thus reducing intracellular mycobacterial survival. To test this, we assessed the efficacy of 2P-AC in reducing Mtb (H37Rv) survival in resting human THP1 macrophages. We observed a significant (p = 0.0365) reduction in bacterial burden at 72 h post-infection of 39% upon treatment with 4 mM 2P-AC compared to the non-treated control (Fig. 8B). Control experiments showed that 2P-AC has no detrimental effect on macrophage viability (Fig. 8B inset). In addition, inhibition of 2P-AC is specific for SapM, as it did not inhibit the other secreted mycobacterial phosphatases MptpA and MptpB when tested at 4 mM concentration. Importantly, 2P-AC showed no effect on extracellular cultures of Mtb at 72 h, therefore the reduction in bacterial burden observed is consistent with the proposed role of SapM in mycobacterial intracellular survival^10,14^. These findings demonstrate that SapM inhibition may be effective in assisting mycobacterial clearance during infection, thus providing a novel mechanism of action for TB treatment.

## Discussion

In this study, we provide new insights into the mechanism of catalysis, substrate specificity and inhibition of SapM. Our findings demonstrate that SapM is an atypical monoester alkaline phosphatase, with a serine-based mechanism of catalysis probably metal-dependent. Particularly relevant to SapM function and pathogenesis, is its activity towards PI(4,5)*P*_2_ and PI3*P*, two PIs that act at the early stages of microbial phagocytosis and phagosome formation. This suggests that SapM may have a pleiotropic role with a much wider impact on Mtb infection than initially thought. Finally, we have identified two inhibitors of SapM, 2P-AC and L-AC, which define two different mechanisms (competitive and metal oxidation) by which the catalytic activity of this phosphatase could be regulated. Critically we demonstrate that 2P-AC reduces mycobacterial survival in macrophage infections.

Our bioinformatics analysis revealed that SapM and orthologues share a conserved phosphoesterase domain with the superfamily of alkaline phosphatases (Fig. 1A). Consistent with this, we have demonstrated that SapM is a monoester phosphatase capable of hydrolysing a broad range of phosphosubstrates including cysteamine *S*-phosphate, an alkaline phosphatase substrate, and ATP for which the enzyme showed the highest specificity (Fig. 5A,B). Based on the multiple sequence alignment and using mutagenesis and activity assays, we have identified for the first time the key catalytic residues of SapM, including the nucleophile S95 and residues (E59, N60, D238 and E239 H160 and H204) which are associated with metal and substrate binding (Fig. 3A,B). Furthermore, we have identified Lys159, as critical for phosphoinositide binding (Fig. 6C,D).

SapM shares a similar mechanism of catalysis with another phosphatase, Ft-AcpA, which is critical for survival of *F*. *tularensis* in host cells. Inhibition of Ft-AcpA with 2P-AC impaired survival of *F*. *tularensis* in infected macrophages^27^. We have demonstrated in this study, that 2P-AC is a competitive inhibitor of SapM (Fig. 7D) with efficacy in reducing mycobacterial burden in infected macrophages, despite having no effect on extracellular bacterial cultures (Fig. 8A,B). We expect that more potent inhibitors, perhaps based on the 2P-AC scaffold, would provide good starting points for drug development and further validation of SapM as a drug target.

Manipulation of the host phosphoinositide metabolism is a common mechanism in microbial pathogenesis to promote colonisation of macrophages during infection^4,5,45,46^. This is particularly relevant for Mtb infections where alteration in PI dynamics affects phagosomal maturation and enables long-term survival of the bacteria in alveolar macrophages. We show in this study that SapM is able to hydrolyse all seven phosphoinositides albeit with higher specificity for PI(4,5)*P*_2_ and PI3*P*, and to our knowledge, SapM is the first bacterial phosphatase to show such a broad PI activity.

**Figure 9:**
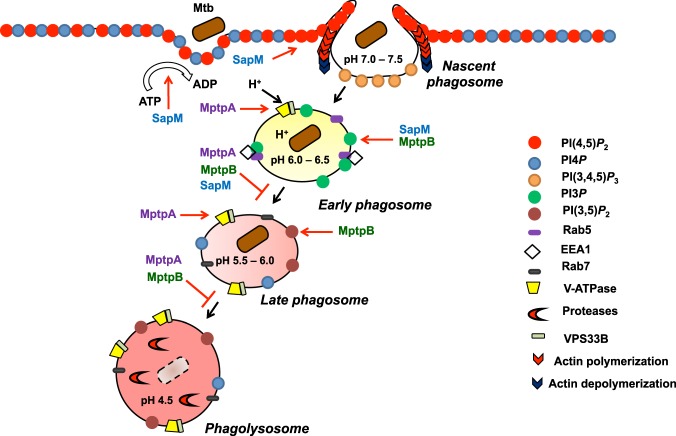
*M*. *tuberculosis* secreted phosphatases regulate phagocytosis and phagosome maturation. After target recognition pseudopodia extension is triggered by an increment of PI(4,5)*P*_2_ in the plasma membrane together with actin polymerization. Sealing of the phagosome is characterised by actin depolymerization and loss of PI(4,5)*P*_2_. SapM may accelerate actin depolymerization by hydrolysing PI(4,5)*P*_2_ to ensure Mtb uptake by the host. The early phagosome is characterised by the presence of Rab5, PI3*P* and EEA1, essential molecules to allow early phagosome-endosome fusion. Hydrolysis of PI3*P* by SapM and MptpB prevents the Rab5-Rab7 exchange that mediates the transition to late phagosome. Phagosomal acidification occurs by acquisition of V-ATPase that uses ATP. SapM may contribute in preventing acidification by hydrolysis of ATP. MptpA inhibits phagosome acidification by hydrolysis of VPS33B and inhibition of V-ATPase trafficking to the late phagosome. MptpB hydrolyses PI(3,5)*P*_2,_ which is required for late phagosome/phagolysosome fusion. The coordinated action of the three secreted phosphatases prevents phagolysosome fusion and pathogen destruction thus promoting Mtb survival in the host.

Previously, another two secreted phosphatases, MptpA and MptpB, have been implicated in the regulation of phagosomal maturation by Mtb^8,47–49^. Based on the current knowledge and our new data on the activity of SapM, we propose a model in which these three mycobacterial phosphatases MptpA, MptpB and SapM, could be acting in a coordinated manner to control phagosome biogenesis, acidification and maturation, blocking phagolysosome fusion and thus promoting survival of Mtb in the macrophage (Fig. 9).

The role of Mtb in phagosome maturation arrest is a complex mechanism where many players take part at different stages^5^ (Fig. 9). Initial phagosome formation and scission from the plasma membrane requires hydrolysis of PI(4,5)*P*_2_^50^. This is a critical step for pathogenic invasion as described for *Salmonella*^51^ and *Yersinia*^52^. In this context, PI(4,5)*P*_2_ hydrolysis by SapM could represent a critical step to facilitate Mtb uptake and colonisation of the host.

The early phagosome membrane is characterised by the presence of PI3*P* critical for phagosomal maturation downstream events^5,9^. Hydrolysis of PI3*P* by SapM has been implicated in phagosome maturation arrest^9^. We have previously reported that MptpB, also dephosphorylates PI3*P*^49^, and that inhibition of MptpB, affects PI3*P* dynamics in infected macrophages^8^. Hence, it is probable that the combined hydrolysis of PI3*P* by both SapM and MptpB enhances maturation arrest at this early stage. MptpB also dephosphorylates PI(3,5)*P*_2_^49^, a key lipid for late phagosome formation and fusion to lysosomes^53^.

Another critical event that promotes Mtb survival is the prevention of phagosome acidification. Acidification is triggered by V-ATPase (proton-pumping ATPase) recruitment, which requires ATP to translocate protons (H^+^) creating the acidic environment inside the phagosome^54^. MptpA inhibits V-ATPase trafficking to the mycobacterial phagosome by dephosphorylation of VPS33B (human vacuolar protein sorting 33B)^47^, thus preventing acidification. Since SapM hydrolyses ATP with high efficiency, this could also contribute to prevent acidification. Therefore, arrest of phagolysosomal fusion and subsequent bacterial destruction may be controlled by the coordinated activities of these three secreted phosphatases to enhance mycobacterial survival in the host (Fig. 9). Testing this model and the spatio-temporal involvement of three Mtb phosphatases in phagosome maturation and PI dynamics will be the focus of future work.

## Material and Methods

### Bioinformatics analysis

BLAST searches in the UniProt Knowledgebase (UniProtKB)^55^, were performed using the full length sequence to identify SapM-related sequences. Next, using InterPro database^56^ conserved domains and families were identified^57^. For the BLAST searches, the E-Threshold was set at two values: 0.0001 and 10. Multiple sequence alignments were performed using Clustal Omega^58^ and manually edited using the program Jalview Version 2.10.3b1^59^.

### Cloning and mutagenesis of SapM

The open reading frame of Rv3310, encoding the SapM gene, was amplified from *Mycobacterium tuberculosis* (Mtb) H37Rv DNA. The amplicon from residues 44 to 299, corresponding to the mature protein^11^ was cloned into pET22b (Novagen) vector using the forward (5′-GCTGCTCCATGGCGAGTGCCCTGCCGACC-3′) and reverse (5′- GCTCGTGCGGCCGCGTCGCCCCAAATATCGGTTATTGG-3′) primers containing NcoI and NotI restriction sites (New England Biolabs, UK) to generate the C-terminal His_6_-tagged SapM construct. Site directed mutagenesis was carried out using Accuzyme^TM^ DNA Polymerase (BIOLINE) for thermal cycling and the PCR product inserted into pET22b vector. The His_6_-tagged SapM construct was used as a template. DNA sequence analyses confirmed that residues E59A, N60A, S95A, K159A, H160A, H204A, D238A and E239A were mutated and that no additional changes were introduced.

### Overexpression and purification of recombinant SapM

Constructs (wild-type and mutants) were transformed into *Escherichia coli* C41 (DE3) strain for expression of recombinant proteins. Single colonies of *E*. *coli* C41 (DE3) were inoculated in 10 ml of LB (Luria-Bertani) broth supplemented with ampicillin (100 µg/ml) and 1% glucose and incubated overnight. Subsequently the overnight culture was diluted into 400 ml of auto-induction media^60^ and grown at 37 °C with shaking (220 rpm) until OD_600_ reached ~0.4. The culture temperature was reduced at 20 °C for protein expression and culture was harvested (6500 g for 15 min at 4 °C) 24 h later. Recombinant SapM (wild-type and mutants) was purified by nickel-affinity chromatography using an AKTA purification system (GE Healthcare). Cell pellets were re-suspended in lysis buffer containing 50 mM HEPES, 500 mM NaCl, with 1 mM ethylenediaminetetraacetic acid (EDTA), 1 mg/ml lysozyme, 1% N-lauroylsarcosine sodium salt (sarkosyl) (Sigma-Aldrich), 38 units/ml of benzonase nuclease (Sigma-Aldrich) and complete EDTA-free protease inhibitor cocktail (Roche), pH 7 and incubated on ice for 30 min. Cells were disrupted by sonication (Sonics Vibra-Cell). Lysates were cleared by centrifugation at 12,000 g for 1 h at 4 °C. The cell lysate was filtered through a 0.45 µm flask filter and loaded onto a 1 ml HisTrap^TM^ HP column (GE Healthcare) in binding buffer containing 50 mM HEPES, 500 mM NaCl and 0.05% sarkosyl, pH 7 and washed with buffer containing 50 mM HEPES, 500 mM NaCl and 0.05% Sarkosyl, and 60 mM imidazole, pH 7. Protein was eluted with a gradient of imidazole (from 60 mM to 250 mM) in the elution buffer. Fractions containing the protein were further purified using a second 1 ml HisTrap^TM^ HP column and eluted with 200 mM imidazole in elution buffer (50 mM HEPES, 500 mM NaCl and 0.05% Sarkosyl). Purity of fractions was verified in a coomassie-stained 10% SDS-PAGE run under reducing conditions with beta-mercaptoethanol. The identity of SapM was confirmed by trypsin digestion and mass spectrometry (LC/MS/MS) using a sample protein excised from an acrylamide gel corresponding to the second IMAC purification. Multiangle light scattering analysis confirmed a molecular mass of 30.3 kDa and that the sample was monodisperse after IMAC purification.

### Overexpression and purification of MptpA and MptpB

The N-terminal histidine tagged constructs for MptpA and MptpB were generated as previously described^7,8^. Constructs were transformed into *E*. *coli* BL21(DE3) and expression induced at 18 °C with 0.5 mM IPTG (isopropyl-β-D-thiolgalactopyranoside) for MptpB and 0.1 mM IPTG for MptpA for 16 h. Purification of both MptpA and MptpB was done by nickel affinity chromatography followed by a size exclusion chromatography using a Superdex75 (10/300) column (GE Healthcare). MptpB and MptpA proteins were eluted in buffer containing 20 mM Tris-Base, 150 mM NaCl, 3 mM EDTA, pH 7.

### Activity assays using p-Nitrophenyl phosphate (pNPP)

Enzymatic activity of SapM was assessed by hydrolysis of *p*-nitrophenyl phosphate *(p*NPP) (Sigma-Aldrich) according to the manufacturer’s protocol. Briefly, we tested pH ranges 4 to 6, using a 150 µl reaction mixture containing 0.5 µg protein, 1 mM *p*NPP in 50 mM Tris-Base, 150 mM sodium acetate with/without 3 mM dithiothreitol (DTT) and pH ranges 7 to 9.5 in 50 mM Tris-Base, 150 mM NaCl and with/without 3 mM DTT. Samples were incubated at 37 °C for 30 min at which time the reaction was quenched by adding 50 µl of NaOH 1 M. The absorbance was subsequently read at 405 nm using a Multiskan® Spectrum spectrophotometer (Thermo Scientific). The concentration of *p*-nitrophenol produced was determined using a *p*-nitrophenol standard curve (15 µM–2000 µM of Sigma-Aldrich *p*-nitrophenol standard solution). Optimal pH was tested using the His_6_-tagged and naked SapM protein (without the tag). The assay was optimized with respect to time and protein concentration so that the reaction is performed within the linear range of the initial velocity.

The effect of metals and inhibitory compounds were tested using a 150 µl reaction mixture containing 1 µg of protein in 50 mM Tris-Base, 150 mM NaCl, pH 7.5 and the different metals or inhibitory compounds (metals and inhibitory compounds at 10 mM, except FeCl_3_ at 1 mM) and incubated for 30 min at room temperature, followed by the addition of *p*NPP to a final concentration of 500 µM and further incubated for 30 min at 37 °C. The reaction was quenched by adding 50 µl of NaOH 1 M followed by the steps explained above. Metals and compounds sodium tartrate (Na-Tr), egtazic acid (EGTA), sodium fluoride (NaF), 2-phospho-L-ascorbic acid (2P-AC), sodium orthovanadate (Na-OV), sodium molybdate (Na-Mb), L-ascorbic acid (L-AC) were all from Sigma-Aldrich. EDTA, (Fisher Scientific) and tetramisole hydrochloride (THC) (Fluorochem).

The phosphatase activity of SapM (wild-type and mutants) was determined using a 150 µl reaction mixture containing 0.5 µg of protein and 1 mM *p*NPP in 50 mM Tris-Base, 150 mM NaCl, pH 7.5 and incubated at 37 °C for 30 min, 60 min and 90 min. The absorbance was read at 405 nm every 30 min after quenching the reaction. Concentration was calculated as explained above. All the experiments were performed in triplicates in 96-well microplates F-bottom clear plates (Greiner bio-one) in at least two separate studies.

### Activity assays using the Malachite Green Assay

The amount of free phosphate during the dephosphorylation of phosphosubstrates was determined with the malachite green assay (Sigma-Aldrich) according to the manufacturer’s protocol. The substrates tested were: phosphoinositide substrates diC8-PI3*P*, diC8-PI(3,4)*P*_2_, diC8-PI(3,5)*P*_2_, diC8-PI4*P*, diC8-PI(4,5)*P*_2_, diC8-PI5*P*, and diC8-PI(3,4,5)*P*_3_ (all from Echelon Bioscience), O-phospho-L-tyrosine (P-Tyr), O-phospho-L-serine (P-Ser), O-phospho-L-threonine (P-Thr), cysteamine *S*-phosphate sodium salt (CysS-P), bis-(*p*-nitrophenyl) phosphate sodium salt (Bis *p*NPP), phosphorylcholine chloride (PC), O-phosphorylethanolamine (PETh), mono-sodium phosphoenolpyruvate hydrate (PEP), glycerophosphate disodium salt hydrate (GlyP), inosine 5′-monophosphate disodium salt hydrate (IMP), guanosine 5′-monophosphate disodium salt hydrate (GMP), guanosine 5′-triphosphate sodium salt hydrate (GTP), adenosine 5′-triphosphate (ATP) disodium salt hydrate, adenosine 5′-diphosphate sodium salt (ADP), adenosine 5′-monophosphate disodium salt (AMP), trehalose 6-phosphate dipotassium salt (T6P), D-fructose 6-phosphate disodium salt hydrate (F6P), phytic acid (PhyA), α-naphthyl phosphate (α-NP), β-nicotinamide adenine dinucleotide 2′-phosphate reduced tetrasodium salt hydrate (NADPH), riboflavin 5′-monophosphate sodium salt hydrate (FMN), adenosine 3′,5′-cyclic monophosphate sodium salt monohydrate (c-AMP), D-glucose 6-phosphate sodium salt (G6P) (all from Sigma-Aldrich), and adenosine 5′-monophosphate sodium salt (AMP) (Fisher Scientific).

The specific activity of SapM towards the 34 substrates was determined using a 100 µl reaction mixture containing reaction buffer (50 mM Tris-Base, 150 mM NaCl, pH 7.5), 80 µM of substrate and 1 µg of protein. The reaction mixtures were incubated at 37 °C for 30 min prior to the addition of 15 µl of malachite green reagent. Subsequently, the reaction was further incubated for 15 min at room temperature (21 °C). The absorbance was read at 620 nm using a Multiskan® Spectrum spectrophotometer (Thermo Scientific). The concentration of free phosphate produced was determined using a phosphate standard curve (25–3000 pmol of Sigma-Aldrich phosphate standard solution).

Specificity activity (wild-type and mutants) towards *p*NPP, ATP, PI3*P* and PI(4,5)*P*_2_ was evaluated with 80 µM of substrate and 0.1 µg of protein. For kinetic measurements (wild-type and mutants), we used 3–750 µM of substrate and 0.1 µg of protein. Incubation was at 37 °C for 1 h. Following steps were the same as explained above. All the experiments were performed in triplicates in 96-well microplates F-bottom clear plates in at least two separate studies.

### Kinetic analysis

The Michaelis-Menten kinetic parameters *V*_max_ and *K*_m_ were calculated using non-linear regression fit in GraphPad Prism 7.01 for Windows by plotting velocity as a function of *p*NPP concentration. The *k*_cat_ was determined by dividing the *V*_max_ by the molar enzyme concentration. Values are calculated as average of at least two separate studies performed in triplicates with ±SEM.

### EPR and ICP-MS studies

Both analyses were performed using a sample containing ~4 mg/ml of protein in 200 mM NaCl, 20 mM HEPES, pH 7 with and without EDTA present. For EPR, continuous‐wave EPR spectra were recorded at 9.4 GHz (X‐band) on a Bruker EMX spectrometer equipped with a Super‐high‐Q rectangular cavity and an Oxford ESR‐900 liquid helium cryostat. Instrument operating conditions were modulation amplitude 4.0 G, modulation frequency 100 kHz, microwave power 0.3 mW, temperature 5.0 K. For ICP-MS, sample was treated with nitric acid 65% and measured in an Agilent 7500 ICP-MS (Agilent technologies, UK). The equipment was fitted with an auto sampler (ASX-500 series), a quartz double-pass spray chamber (Peltier-cooled, Scott-type), a concentric MicroMist nebuliser and a third generation Octopole Reaction System (ORS3). This allows for the correction of spectral interferences by the addition of He or H_2_ gas to the collision cell, producing ions of a lower kinetic energy that enable the analyte ions with a higher energy than the multipole to be transmitted to the mass analyser. The digested sample solution was added via a T-piece before nebulisation, along with the internal standard solution. Ion standards are made up from single stocks and mixed element standards supplied by VWR and Johnson Matthey.

### Inhibition assays

The IC_50_ of 2-phospho-L-ascorbic acid (2P-AC) and L-ascorbic acid (L-AC) was determined using a 150 µl reaction mixture containing 0.5 µg of protein in 50 mM Tris-Base, 150 mM NaCl, pH 7.5 and the different inhibitors (0.02 µM–20 mM). The reaction was incubated for 30 min at room temperature, followed by the addition of *p*NPP to a final concentration equal to its *K*_m_ value and incubated for 30 min at 37 °C. The reaction was quenched by adding 50 µl of NaOH 1 M to measure absorbance at 405 nm. The concentration of *p*-nitrophenol produced was determined using a *p*-nitrophenol standard curve and expressed as a percentage of the specific activity. Data was plotted as a function of log inhibitor concentration to calculate the IC_50_ using a four-parameter dose-response curve in GraphPad Prism 7.01. Selectivity assays for MptpA and MptpB were done by determining specific activity towards *p*NPP in the presence of 4 mM 2P-AC in a reaction assay containing 5 µg of protein, 50 mM Tris-Base, 50 mM Bis-Tris, 100 mM Sodium Acetate, pH 6 and incubating for 30 min at room temperature.

To determine the type of inhibition of 2P-AC and L-AC, different inhibitor concentrations were used (for L-AC 146 µM, 243 µM and 365 µM; for 2P-AC 140 µM, 234 µM and 351 µM) in a 150 µl reaction mixture containing 0.5 µg of protein in 50 mM Tris-Base, 150 mM NaCl, pH 7.5. Samples were incubated for 30 min at room temperature, followed by addition of different concentrations of *p*NPP (62.5 µM, 125 µM, 250 µM, 500 µM, 1 mM and 2 mM) and further incubated for 30 min at 37 °C. The reaction was quenched by adding 50 µl of NaOH 1 M. Velocity (*V*) was plotted as a function of *p*NPP concentration and fit in a Lineweaver-Burk plot (double-reciprocal) using GraphPad Prism 7.01. All the experiments were performed in triplicates in at least two separate studies.

### Cell and bacterial culture conditions

THP1 macrophages cells (ATCC) were cultured in Roswell Park Memorial Institute-1640 medium (RPMI-1640) (R8758-Sigma-Aldrich) containing L-glutamine supplemented with 10% heat inactivated fetal bovine serum (FBS, Invitrogen) at 37 °C in a 5% CO_2_. *M*. *tuberculosis* (Mtb) laboratory strain H37Rv was grown on Middlebrook 7H9 broth (BD Diagnostics) at 37 °C in 5% CO_2_ or Middlebrook 7H10 agar (both mediums were supplemented with 0.05% Tween 80, 0.2% glycerol and 10% OADC). Mtb cultures were prepared using 1 ml of mid-log phase stock into 20 ml of fresh media and incubated static for 6 days prior to being used in infection assays or inhibitory assays. All experiments with Mtb were carried out in a biosafety level 3 containment facility.

### Extracellular growth of *Mycobacterium tuberculosis* and macrophage infections

2-phospho-L-ascorbic acid (2P-AC) and L-ascorbic acid (L-AC) effect on extracellular cultures of Mtb was assessed by adding 1 mM or 4 mM of the compounds in 20 ml of Middlebrook 7H9 broth. Compounds were added to the culture in day 0 and 1 and allowed to grow over 11 days. Mycobacterial growth was monitored by optical density (OD) at 600 nm. Experiments were performed in triplicates in at least two separate studies. For statistical analyses, OD values were evaluated by two-way ANOVA followed by a multiple comparison analyses of variance by Bonferroni test (GraphPad Prism 7.01 for Windows). Differences were considered significant at the 95% level of confidence.

For the macrophage infections, THP1 cells were seeded in 24-well culture plates (Corning) at a density of 1 × 10^5^ cells per well (in 500 µl media) and treated with 200 nM PMA for 2 h. After treatment, media was changed for fresh RPMI (with 10% FBS) overnight. The following day, media was removed and cells were washed with PBS and 500 µl of fresh RPMI was added containing 2P-AC (1 mM and 4 mM dissolved in water) before infecting the cells with Mtb at MOI of 5:1 (bacteria:macrophage). After 4 h of infection, THP1 cells were washed three times with Dulbecco’s PBS and fresh RPMI was added containing 2P-AC. After 24 h the media was removed and cells were washed twice with PBS prior to the addition of RPMI containing 2P-AC. At 72 h post infection cells were lysed with ice-cold water and plated onto 7H10 agar. Colonies were counted after 14 days. All experimental points were plated as 10-fold dilutions in triplicates. Each sample was setup in triplicates in at least two independent experiments. For statistical analyses, CFU/ml values were evaluated by one-way ANOVA followed by a multiple comparison analyses of variance by Bonferroni test (GraphPad Prism 7.01 for Windows). Differences were considered significant at the 95% level of confidence. All experiments with Mtb were carried out in a biosafety level 3 containment facility.

### Cytotoxicity assays

MTT assay was performed as described previously^61^. Briefly, THP1 cells were seeded in 96-well culture plates (Corning) at a density of 5 × 10^4^ (in 200 μl media) and treated with 200 nM PMA for 2 h. After treatment, media was changed for fresh RPMI (with 1% FBS) and leaved it overnight. The following day media was removed and fresh RPMI was added supplemented with 2P-AC inhibitor. Inhibitor was added to each well at 0 h and 24 h. After 48 h, cells were washed once with PBS and 200 μl of fresh RPMI was added. At 72 h, media was removed and changed for 200 μl of fresh RPMI. Cell viability was assessed by adding 50 µl of filter sterilised MTT (5 mg/ml in PBS) to each well followed by a 2 h incubation period at 37 °C in a 5% CO_2_. Media was removed and the blue formazan crystals trapped in cells were dissolved by adding 200 µl of DMSO and 25 µl of Sorensen’s glycine buffer. The absorbance at 570 nm was measured in a plate reader. Each sample was setup in triplicates in at least two independent experiments.

## Supplementary information

Supplementary information
