# Supplementary material for: Mechanism of catalysis and inhibition of Mycobacterium tuberculosis SapM, implications for the development of novel antivirulence drugs
Source: Sci Rep. 2019 Jul 16;9:10315. doi: 10.1038/s41598-019-46731-6 (PMC6635428; doi:10.1038/s41598-019-46731-6)
Supplement: Supplementary file 1 — Supplementary information [file 41598_2019_46731_MOESM1_ESM.pdf]

Mechanism of catalysis and inhibition of *Mycobacterium tuberculosis* SapM, implications for the development of novel antivirulence drugs.

Paulina Fernandez-Soto[1], Alexander J. E. Bruce[1], Alistair J. Fielding[2], Jennifer S. Cavet[1] and Lydia Tabernero[1,*].

1. School of Biological Sciences, Faculty of Biology Medicine and Health, University of Manchester, Manchester Academic Health Science Centre, Manchester, M13 9PT, UK.
2. Pharmacy and Biomolecular Sciences, Liverpool John Moores University, James Parsons Building, Byrom Street, Liverpool, L3 3AF.

* corresponding author: Lydia Tabernero (Lydia.Tabernero@manchester.ac.uk)

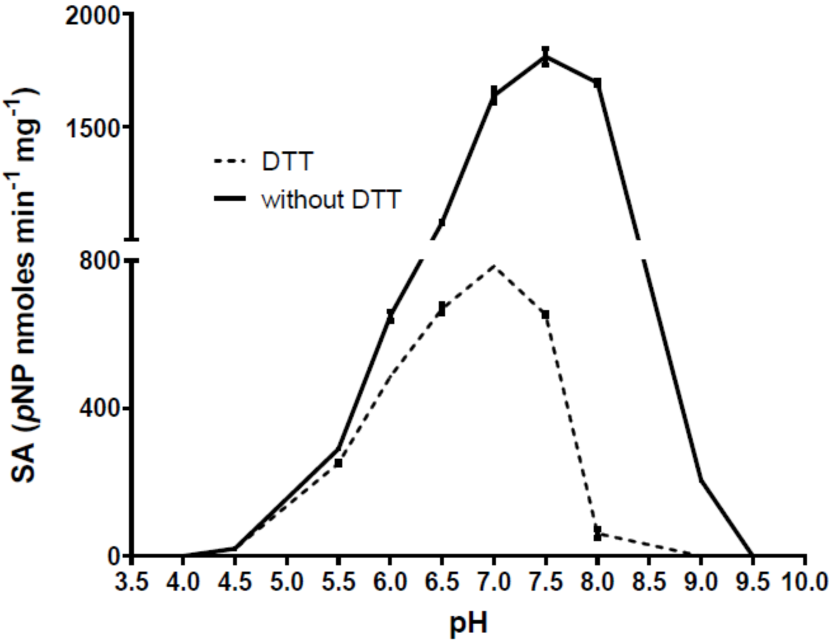

Supplementary figure 1. Enzymatic activity of tag-less SapM. Enzymatic activity of His_6-less SapM protein was assessed using *p*-Nitrophenyl phosphate (*p*NPP) assay. Hydrolysis of *p*NPP was measured at pH values ranging from 4 to 9.5 in the presence and absence of 3 mM dithiothreitol (DTT). Specific activity (SA) was calculated as nanomoles of *p*-nitrophenol (*p*NP) released per mg of protein and min of the reaction. Optimal activity is observed at pH 7.5 in absence of DTT. Error bars represent standard deviation of the mean (SD) of triplicates.

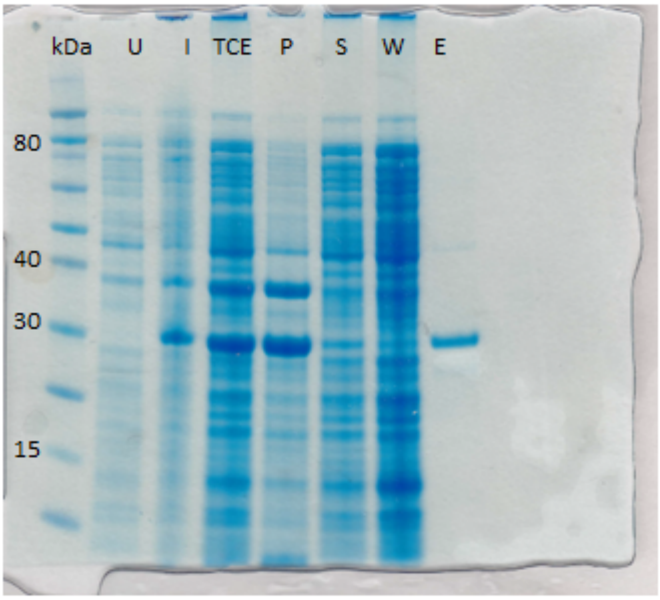

Supplementary figure 2. Coomassie stained 10% SDS-PAGE full-length gel shows expression and purification of recombinant His$_6$-SapM (Mw 28.8 kDa) that appears as a single band (lane E). Solubilisation was achieved using 1% sarkosyl in the lysis buffer. Purification was done by nickel-affinity chromatography and eluted with 200 mM imidazole. U: uninduced, I: induced, TCE: total cell extract, P: pellet, S: soluble, W: wash, and E: elution
